# Testing a chain mediation model of effort-reward imbalance, Confucian values, job satisfaction, and intention to quit among Chinese vocational education teachers

**DOI:** 10.3389/fpsyg.2023.1341928

**Published:** 2024-01-12

**Authors:** Guantao Wang, Jinyu Shi

**Affiliations:** ^1^Tourism Management, School of Culture and Tourism, Chongqing City Management College, Chongqing, China; ^2^Department of Social Work, School of Civil Affairs and Social Governance, Chongqing City Management College, Chongqing, China

**Keywords:** Confucian values, vocational education, resignation consideration, societal dynamics in China, occupational strain, influence of tradition

## Abstract

**Context:**

This research delves into the significant impact of Confucian Values on the societal fabric of China, particularly in the realm of vocational education. In this setting, these principles are instrumental in guiding both educators and students. The study employs the Culture-Value Structure (CVS) model to dissect the intricate dynamics between Confucian Values, the Effort-Reward Imbalance (ERI), educators’ job satisfaction, and their inclination toward leaving the profession. Teachers in vocational education, who are often regarded as moral guides, play a pivotal role in the ethical and social upbringing of students. Adopting Confucian values not only promotes academic success but also nurtures all-round development, equipping students for conscientious societal roles. In the backdrop of Confucian influence, these educators face substantial stressors in the workplace due to varied demands. The ERI model, proposed by Siegrist, serves as a tool to comprehend the stress experienced when there is a disproportion between efforts and rewards. This study delves into how ERI correlates with job satisfaction among vocational education teachers, with a focus on the modifying effect of Confucian values. Additionally, it examines the potential role of job satisfaction in mediating the relationship between ERI and the tendency to consider leaving the job. The research illuminates the complex interrelation of cultural values, occupational stress, job contentment, and career decision-making in the context of vocational education in China.

**Methodology:**

The research involved a group of 332 Vocational education teachers from diverse Chinese institutions. Through thorough statistical analysis, the study validated the model’s effectiveness, notably indicating a substantial direct impact of ERI on the Intention to Quit.

**Findings:**

The investigation pinpointed Factors I (Integrity), II (Confucian Ethos), and IV (Moderation) as key determinants of job satisfaction. Notably, an increase in job satisfaction was found to inversely relate to the likelihood of leaving the profession, implying it could lessen the propensity to resign. The research applied a Chain Mediation Model to elucidate the influence of ERI on the decision to quit, mediated by various factors. The findings highlight the complex interaction of elements influencing teachers’ decisions to leave, showcasing the utility of sophisticated statistical methodologies in decoding complex social dynamics.

## Introduction

The pervasive influence of Confucian Values in Chinese society, particularly in educational settings, forms the cornerstone of this research. While these principles provide a moral and ethical framework for educators and students alike, there is a notable gap in understanding how these values intersect with contemporary workplace stressors, especially within vocational education. The CVS model, as proposed by Schwartz and Bardi in 2001, categorizes Confucian Values effectively, yet its application in understanding vocational education dynamics remains underexplored (Schwartz and Bardi, 2001).

This article addresses this gap by examining the interplay between Confucian Values and the Effort-Reward Imbalance (ERI) model, introduced by Siegrist in 1996, within the context of job satisfaction and commitment among Chinese vocational education instructors. While vocational educators in China are often regarded as ethical guides, shaping the comprehensive development of students, they also encounter significant challenges and stress factors in their professional roles. The ERI model provides a lens to understand these stresses, suggesting that imbalances between effort and reward can lead to occupational stress (Siegrist, 1996).

Our research seeks to unravel how Confucian values may mediate the relationship between ERI and job satisfaction in this unique setting. Additionally, we aim to explore the role of job satisfaction as a link between ERI and educators’ commitment to their profession. The primary goal of this study is to shed light on the complex connections between cultural values, occupational stressors, job satisfaction, and dedication to vocational education in China.

Through this exploration, we intend to contribute to a deeper understanding of the multifaceted relationship between Confucian values, workplace dynamics, and the commitment of vocational education instructors to their profession, thereby filling a significant gap in the current research landscape.

### Literature review and hypotheses development

#### Confucian values in the workplace: a global perspective

Confucian values, deeply ingrained in the fabric of Chinese society, have significantly influenced various aspects of life in China. From shaping personal relationships and household financial behavior to influencing workplace dynamics, these values offer a rich tapestry of ethical and moral guidelines. In the education sector, especially in vocational training, Confucian values influence numerous aspects including marital relationships (Cheng, 2021), household financial decisions regarding risky assets (Ge et al., 2023), and workplace dynamics (Liu et al., 2021; Wu et al., 2021; Jiang et al., 2022). Confucian Values guide the interactions between educators and students, molding the educational environment and ethos (Lim and Thien, 2020; Wang, 2023). The CVS model by Schwartz and Bardi (2001) provides a useful framework for understanding these values, categorizing them into four main groups that offer a detailed perspective on their influence, particularly in vocational education.

Integrity and Tolerance: This category, encompassing values like Filial Piety, Righteousness, and Integrity, highlights the respect and obedience toward elders and authority figures, emphasizing harmony in family and society (Ho, 1994). In educational settings, these values translate into a deep respect for teachers and a commitment to learning, where educators are seen as moral exemplars imparting not only knowledge but also ethical values to students. The enduring relevance of these values in modern education systems, such as the emphasis on respectful student-teacher relationships and the cultivation of a moral and ethical learning environment, is notable (Wang, 2023).

Confucian Ethos: Including Benevolence (Ren), and Harmony (He), this group underscores the importance of nurturing positive interpersonal relationships and promoting social unity (Chen et al., 2018). In the context of vocational education, these values are reflected in the empathetic and compassionate approach of educators, fostering a supportive and cohesive classroom atmosphere. This ethos is critical in creating an educational environment where students feel valued and supported, thereby enhancing learning outcomes (Luo et al., 2019).

Loyalty to Ideals and Humanity: Encompassing values like Loyalty (Zhong), and Ritual Propriety (Li), this category underscores adherence to principles and societal norms. Vocational educators, in this context, are seen as paragons of loyalty to educational ideals and are responsible for instilling a sense of ritual propriety in their students, which is crucial for maintaining discipline and order in the learning environment (Yim et al., 2011).

Moderation and Moral Discipline: The final category includes Wisdom (Zhi), promoting careful judgment and self-restraint. In vocational education, this is manifested in teachers balancing academic rigor with moral development, a crucial aspect of holistic education (Yim et al., 2013; Zhou, 2022).

The global relevance of these Confucian values, despite their roots in Chinese culture, is significant. The principles of respect, compassion, loyalty, and wisdom transcend cultural boundaries, offering a universally applicable framework for understanding and improving workplace dynamics and educational practices. In an increasingly interconnected world, these values can provide valuable insights into fostering a harmonious and productive work environment, enhancing employee satisfaction, and improving educational outcomes.

As educational and organizational landscapes evolve globally, integrating Confucian values can lead to the development of more holistic and culturally sensitive approaches. This can be particularly beneficial in multicultural settings, where understanding and aligning with diverse cultural values is key to effective management and educational success. By adapting these timeless principles to contemporary settings, organizations and educational institutions can nurture responsible, ethical, and well-rounded individuals, equipped to thrive in a diverse and dynamic global society.

In conclusion, while rooted in Chinese culture, Confucian values offer a rich source of ethical and moral guidance that is relevant and applicable globally. Their integration into modern educational and organizational practices can provide a foundation for nurturing harmonious, respectful, and productive environments, both in educational settings and in the workplace. Acknowledging and adapting these values in diverse cultural contexts can lead to more effective and culturally responsive management practices, contributing significantly to global educational and organizational success.

### Assessing stress dynamics in vocational education: the application of the effort-reward imbalance model

The concept of stress among vocational education teachers in China, viewed through the lens of the ERI model, presents a significant area of study in organizational behavior. The ERI model, introduced by Siegrist in 1996, offers a theoretical foundation for understanding the relationship between the demands of a job and the rewards received. This model is particularly relevant in the context of vocational education teachers, who are known to experience distinct challenges and high stress levels.

In the domain of vocational education, the notion of “effort” encompasses a range of responsibilities and challenges. These include the emotional labor of nurturing and mentoring young learners, extensive lesson planning, and managing complex classroom dynamics. Vocational education teachers are charged with the integral role of promoting both the intellectual and emotional growth of their students, a role that demands considerable physical and psychological investment.

The “reward” aspect in the ERI model is not limited to financial compensation. For vocational education teachers, rewards also encompass job security, recognition of their efforts, and opportunities for professional development. These forms of acknowledgment and institutional support are crucial for their professional satisfaction and growth. An imbalance, where the efforts of teachers are not matched with adequate rewards, can lead to heightened stress levels.

Empirical research supports the connection between the Effort-Reward Imbalance and various work-related outcomes (Tsutsumi and Kawakami, 2004). In the context of vocational education, this imbalance significantly impacts job satisfaction and the intention to quit. Job satisfaction is a critical psychological state reflecting an employee’s overall evaluation of their job and is crucial for motivation, commitment, and well-being. For vocational education teachers, job satisfaction significantly influences their dedication to their role and organization.

The intention to quit, conversely, reflects an employee’s predisposition to leave their job voluntarily. It is a key indicator of job dissatisfaction and a predictor of actual turnover. In vocational education, a teacher’s intention to quit can adversely affect the quality of education and the stability of the educational workforce.

Within this framework, the study proposes two hypotheses:

*H1*: The Effort-Reward Imbalance experienced by vocational educators will predict their Job Satisfaction.

*H2*: The Effort-Reward Imbalance experienced by vocational educators will predict their Intention to Quit.

These hypotheses aim to elucidate the relationship between vocational educators’ work-related stress, job satisfaction, and their propensity to leave their jobs, thereby contributing to a more comprehensive understanding of the occupational challenges faced by vocational education teachers in China.

#### Mediating role of job satisfaction in the link between effort-reward imbalance and teachers’ intention to quit in vocational education

The exploration of how job satisfaction acts as a mediator in the relationship between vocational education teachers’ stress and their intention to quit is a critical aspect of understanding workplace dynamics within the educational sector in China. The ERI model serves as a foundational framework for analyzing the sources and implications of stress experienced by these educators.

The ERI model elucidates that the stress experienced by vocational education teachers, primarily due to the imbalance between their efforts and rewards, can significantly impact their job satisfaction. Previous research has underscored the mediating role of job satisfaction in linking effort-reward imbalance with various work-related outcomes, including job burnout (Devonish, 2018). In the context of vocational education, when teachers perceive a lack of adequate acknowledgment or feel overwhelmed due to disproportionate efforts relative to their rewards, it invariably leads to a decline in their job satisfaction.

This decline in job satisfaction is intrinsically linked to an increased intention to leave the profession. As Tett and Meyer have highlighted, vocational education teachers who are dissatisfied with their jobs are more likely to consider quitting (Tett and Meyer, 1993). They might seek alternative employment opportunities that promise better conditions or rewards. Consequently, job satisfaction emerges as a critical mediator, conveying the effects of stress, particularly that stemming from effort-reward imbalance, on teachers’ intentions to leave their profession.

In summary, the ERI model provides profound insights into the stressors affecting vocational education teachers in China and their subsequent impact on job satisfaction. This understanding is crucial for fostering an environment that promotes job satisfaction, thereby influencing teachers’ intentions to remain in their profession. In this avenue, the research that provides empirical foundation for this relationships is constantly increasing (Omansky et al., 2016; Ge et al., 2021; Chen et al., 2022; Heming et al., 2023; Tripathi et al., 2023). Addressing the aspects of effort-reward imbalance and enhancing job satisfaction is imperative for retaining competent educators and ensuring the continued excellence of vocational education.

Based on this understanding, the present study proposes the following hypothesis:

*H3*: Job satisfaction will mediate the relationship between Effort-reward imbalance and Intention to Quit among vocational education teachers.

This hypothesis aims to investigate the chain mediation effect of job satisfaction, exploring how it transmits the impact of effort-reward imbalance on the intention to quit, which is a key factor in understanding the dynamics of the teaching workforce in vocational education in China.

#### Mediating influence of Confucian ethics in vocational education

In the realm of vocational education in China, the pervasive and significant influence of Confucian values necessitates an exploration of their mediating role in the dynamic interplay between Effort-Reward Imbalance (ERI), job satisfaction, and the commitment to continue teaching. This intersection of Confucian Values with modern theories of workplace stress offers a novel perspective to understand how deep-seated cultural values shape the professional experiences of vocational education teachers (Li et al., 2005; Lim and Thien, 2020).

Rooted in Chinese culture, Confucian values emphasize virtues like integrity, benevolence, and harmony. These principles could be pivotal in mediating the effects of ERI on job satisfaction. This perspective allows an understanding of how these enduring cultural norms may impact educators’ responses to stress in the workplace. For example, when vocational teachers experience a disparity between their efforts and the rewards they receive, Confucian tenets such as integrity might encourage them to persevere in their roles, driven by a sense of moral responsibility to educate and guide students (Wang and Cheng, 2010). Similarly, a dedication to benevolence could lead educators to focus on their students’ well-being and development, which might enhance their job satisfaction, even under stressful conditions (Wang et al., 2015).

Considering these insights, the following hypothesis is proposed:

*H4*: Confucian Values will serve as a mediating factor in the relationship between Effort-Reward Imbalance and Job Satisfaction in vocational education settings.

Confucian values also potentially mediate the relationship between Effort-Reward Imbalance (ERI) and vocational education professionals’ commitment to their teaching careers. Central to these values is the concept of loyalty to educational ideals, which may inspire educators to maintain their dedication to teaching despite stressors associated with ERI. Additionally, the principle of ritual propriety, or Li, could motivate teachers to uphold professional standards and demeanor, even in the face of challenging situations (Wu et al., 2021).

Furthermore, Confucianism places significant emphasis on wisdom and moderation, qualities that could play a crucial role in helping teachers manage stress stemming from ERI. The virtue of wisdom, or Zhi, advocates for thoughtful decision-making and self-regulation, fostering the development of effective coping mechanisms. Similarly, the principle of moderation encourages a balanced approach to managing professional demands and personal well-being, potentially reducing the adverse impact of ERI on teachers’ inclination to leave their profession (Wang and Wang, 2021).

Based on these observations, the following hypothesis is posited for the study:

*H5:* Confucian Values will act as mediators in the connection between Effort-Reward Imbalance and the Intention to Discontinue Teaching.

**Figure 1:**
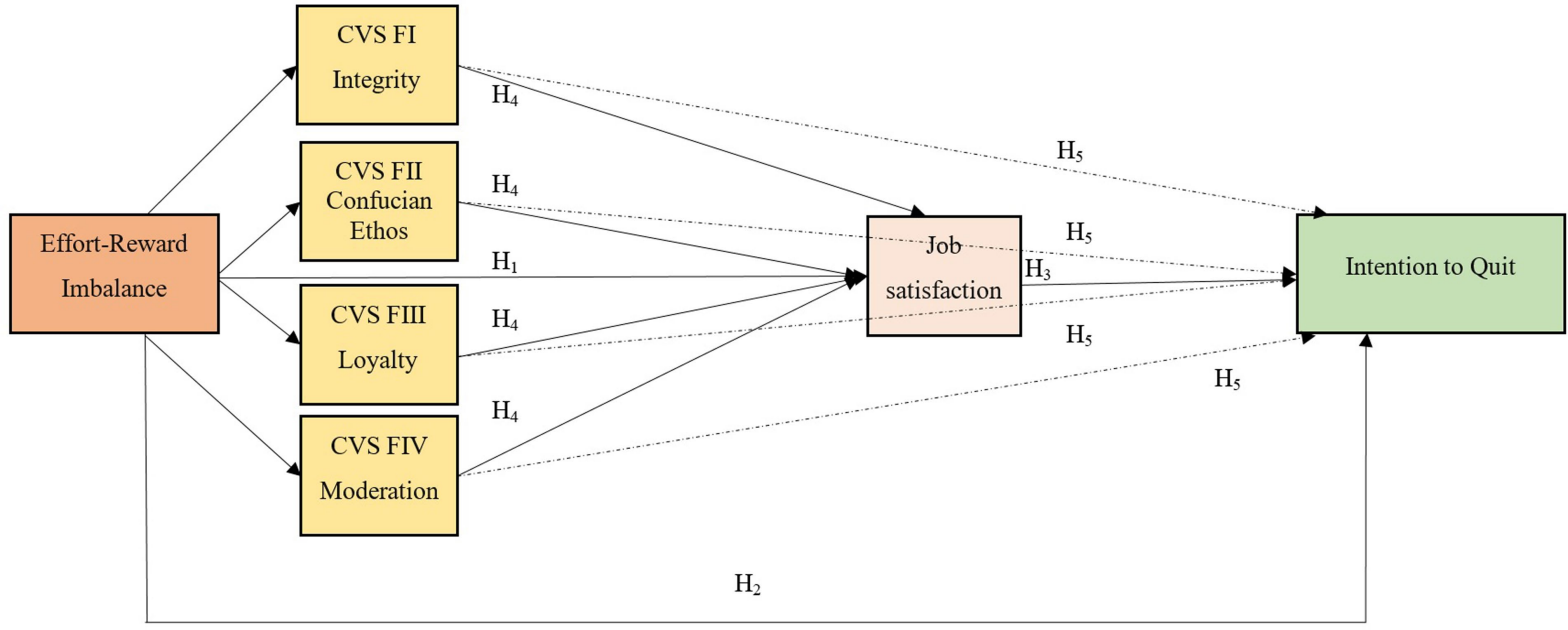
Research model for the present study. Indirect mediation effects illustrated by dotted lines.

The comprehensive model illustrating these relationships is presented in Figure 1.

## Methodology

### Participant selection and data collection process

This study engaged 332 educators from various Chinese vocational education institutions, encompassing Secondary Vocational Schools, Vocational Colleges, Technical Schools, Apprenticeship Programs, as well as Online and Distance Learning platforms, and Government Training Centers. The participant recruitment strategy involved a dual approach, utilizing both email and social media channels. Key platforms such as WeChat, Sina Weibo, QQ, WeChat Moments, and Zhihu were employed to reach potential respondents. Educators were invited to partake in a research study pertinent to their field, with an accompanying link to an online survey. The research protocol received approval from the Ethics Committee of Chongqing City Management College, ensuring compliance with the rigorous ethical standards required for scholarly research. The average age of participants was found to be 36.92 years, with a standard deviation of 9.25, indicating a relatively young cohort. This was reflected in their average tenure in the teaching profession, which stood at 10.67 years with a standard deviation of 9.41. The proportion of female participants was 68.9%.

In our study, we utilized a convenience sampling method to gather data from Chinese vocational education instructors. This approach was adopted due to practical constraints and the accessibility of participants. In convenience sampling, participants are selected based on their availability and willingness to participate, rather than using a random selection process. While this method facilitated the efficient collection of data, we are cognizant of its limitations, particularly in terms of potential sampling bias and the challenges it poses to the generalizability of our findings. To mitigate these limitations, we ensured that our sample included a diverse range of instructors in terms of age, gender, and teaching experience. Despite these efforts, it is important to acknowledge that convenience sampling may not fully represent the broader population of vocational education instructors. This consideration will be taken into account in the interpretation of our study’s results and in the conclusions drawn.

### Informed consent process

Prior to initiating the survey, educators were required to review and consent to a detailed informed consent document. This document outlined the scope and objectives of the study, emphasizing the voluntary nature of participation. It assured complete anonymity for participants, detailed the procedure for data aggregation, and highlighted the right of participants to withdraw at any time without repercussions. Consent was obtained through a digital acknowledgment of understanding these terms. To preserve participant anonymity, demographic information collected was restricted to age, gender, and organizational seniority.

### Measurement tools

#### Effort-reward imbalance instrument

This study utilized the Chinese adaptation of the ERI questionnaire, originally developed Li and colleagues (Li et al., 2005). The questionnaire comprises three principal dimensions: extrinsic effort, reward, and overcommitment. Extrinsic effort is gauged through six items, and reward is assessed via eleven items. Participants rated these items on a 5-point scale, with 1 representing the absence and 5 indicating a very high level of stressful experience. A higher score in the extrinsic effort dimension indicates increased effort demands, whereas a higher score in the reward dimension reflects greater perceived rewards in the work environment. The overcommitment dimension, with six items, utilized a 4-point scale ranging from complete disagreement (1) to complete agreement (4). A higher overcommitment score signifies an excessive work-related commitment. The effort-reward imbalance is measured by the ratio of extrinsic effort to reward, adjusted for the number of items in each scale. This ratio, a method established by Siegrist (1996), indicates the imbalance between effort and reward, where higher values suggest a more stressful work environment. This methodology facilitates an in-depth evaluation of ERI in the context of Chinese culture and language (Wang et al., 2015).

#### Job satisfaction measurement

The study measured job satisfaction using the General Satisfaction subscale of the Chinese Version of the Indiana Job Satisfaction Scale (CV-IJSS) as detailed by Tsang and Wong (2005). This subscale includes 15 items under Factor I: General Satisfaction. Participants rated each item on a scale from 1 (“strongly agree”) to 4 (“strongly disagree”). The subscale demonstrated a Cronbach’s alpha of 0.79.

#### Intention to quit assessment

The Turnover Intention Questionnaire (TIQ), as translated and revised by Lee and Lee (2000), was employed to assess participants’ intention to quit. The TIQ consists of six single-choice questions probing the respondents’ intentions to leave their current profession. Higher scores indicate a stronger intention to quit. This instrument has been effectively used in prior research on different professional groups (Chen et al., 2018), with a Cronbach’s alpha of 0.75.

#### Confucian values instrument

The Chinese Value Survey (CVS), devised by Bond and colleagues, was used to evaluate Confucian values. Recognized for its reliability and validity in social science research, especially in the context of Chinese cultural values, the CVS is divided into several factors. Factor I: Integrity and Tolerance (Cronbach’s alpha 0.81), Factor II: Confucian Ethos (Cronbach’s alpha 0.75), Factor III: Loyalty to Ideals and Humanity (Cronbach’s alpha 0.79), and Factor IV: Moderation and Moral Discipline (Cronbach’s alpha 0.67) are among these. While the reliability value of the fourth factor in our study did not achieve the desired benchmark, it is important to note the scarcity of alternative validated questionnaires specifically designed to assess the Confucian Value model. In light of this, our research team made a conscientious decision to include this factor in our results. We have done so with an understanding of its limitations and with a commitment to provide a transparent and thorough discussion of these limitations. This decision was driven by the recognition that even with its limitations, the inclusion of the fourth factor offers valuable insights into the Confucian Value model, contributing to the broader discourse in this field. Furthermore, we believe that acknowledging and critically examining these limitations can serve as a catalyst for future research efforts aimed at developing more robust and reliable instruments for assessing Confucian values in similar studies. Examples of values in these factors include Persistence, Loyalty to Superiors, Observance of Rituals, and Sense of Cultural Superiority, respectively.

The scales, including those measuring Confucian values and the Effort-Reward Imbalance (ERI), were carefully chosen based on their established validity and reliability in similar demographic contexts. Prior to their application, these scales underwent a rigorous assessment process to confirm their relevance and accuracy for the age group of vocational education instructors in China. This involved reviewing existing literature where these scales were previously applied to similar age groups and consulting with experts in educational psychology and vocational training. Additionally, a pilot test was conducted with a sample representative of our study’s demographic to further ascertain the scales’ suitability and to make necessary adjustments for age-specific nuances. This ensured that our measurement tools were not only theoretically sound but also practically relevant to the age group under investigation.

### Statistical procedures

The data analysis commenced with an initial phase of descriptive statistics to establish baseline characteristics and Pearson’s correlation analysis to explore the relationships between variables. These preliminary analyses were conducted using SPSS version 22.0, a widely recognized statistical software in social sciences research.

Following this, the study employed a more intricate analytical technique, specifically employing a chain mediation model. This approach was crucial for a detailed investigation of the mediating influences of Confucian values and job satisfaction on the relationship between effort-reward imbalance (ERI) and the intention to quit. The chain mediation analysis was carried out using Model 80 within Hayes’ Process Macro (Hayes, 2022). This advanced statistical tool is specifically designed to dissect and understand complex mediation relationships in social science research, offering robust capabilities for handling multiple mediators in a single model.

The chain mediation model is particularly suited for unraveling sequential mediation processes, where one variable mediates the relationship between two other variables and then itself becomes a predictor for a subsequent mediator. In the context of our research, this model facilitated a layered analysis where we first assessed how Confucian values might mediate the effect of ERI on job satisfaction. Subsequently, we explored how this transformed job satisfaction could further influence the relationship between Confucian values and the participants’ intention to quit. This sequential mediation process allows for a nuanced understanding of how multiple factors interact in a cascading effect, ultimately impacting the outcome variable.

To ensure the robustness of our findings, we employed bootstrapping methods with 5,000 resamples, a technique recommended for testing indirect effects in mediation models. This method provides a more reliable estimate of the mediation effect and its confidence intervals, reducing the likelihood of Type I errors. Moreover, we conducted sensitivity analyses to assess the stability of our results under different model specifications and variable coding’s, further ensuring the validity and reliability of our conclusions.

In summary, the methodological approach adopted in this study combines descriptive statistics, correlation analysis, and advanced chain mediation modeling to provide a comprehensive understanding of the intricate relationships among ERI, Confucian values, job satisfaction, and intention to quit. These statistical procedures are meticulously chosen to address the research objectives and to offer a deep insight into the dynamics under study.

## Results

### Mediated chain model

**Figure 2:**
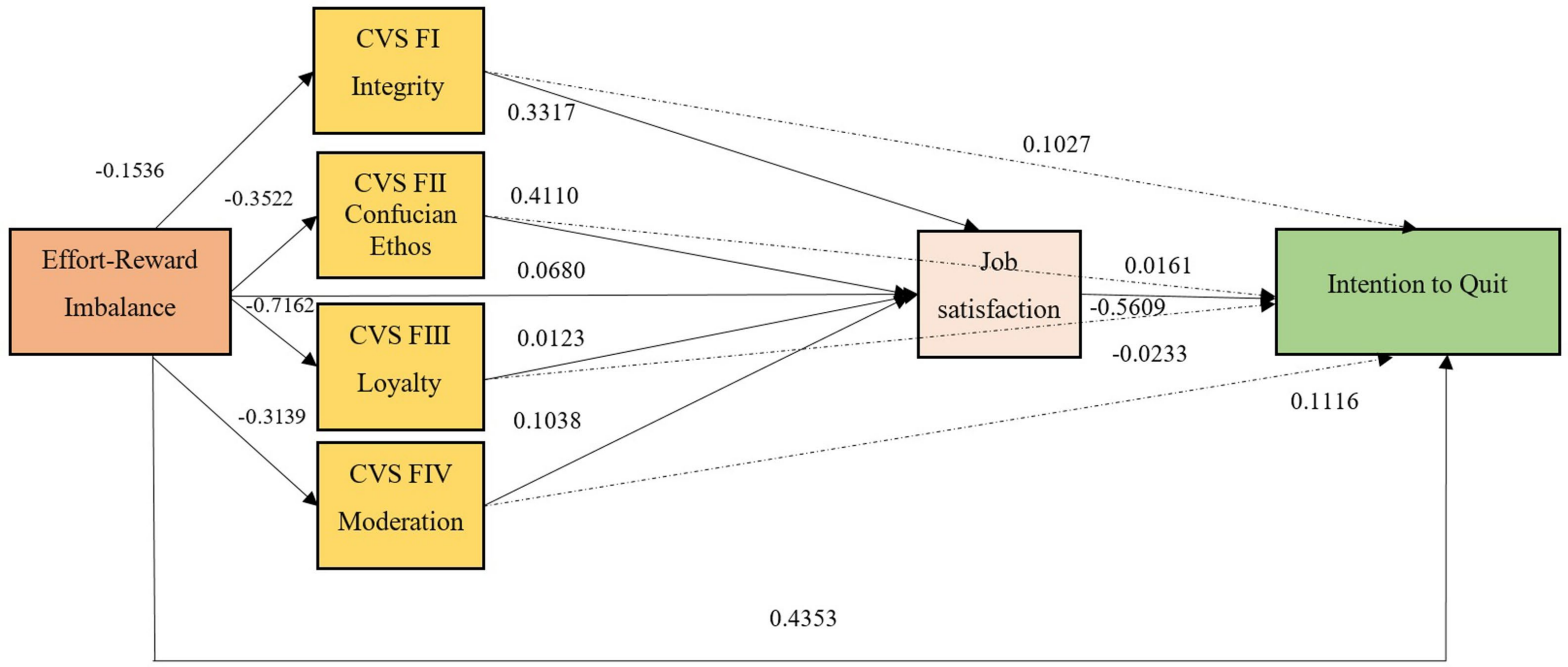
Regression coefficients of the variables in the research model. Indirect mediation effects illustrated by dotted lines.

The statistical parameters of the chain mediation model, encompassing *R* = 0.4957, R-squared = 0.2457, Mean Squared Error = 10.8350, *F*-value = 107.4748, degrees of freedom 1 (df1) = 1, degrees of freedom 2 (df2) = 330, and a value of *p* of 0.0000, corroborated the strength and validity of the baseline model. Figure 2 illustrates these results, asserting the reliability of both the model and its derived conclusions for interpretative purposes.

The findings from the analyses executed using the PROCESS procedure revealed a significant direct effect of ERI on the Intention to Quit, evidenced by a coefficient of 0.1675 and a value of *p* of less than 0.0001. This indicates that Effort-Reward Imbalance exerts a considerable direct influence on the Intention to Quit, even when factoring in the mediating variables. Moreover, the total effect of ERI on the Intention to Quit was also significant, with a coefficient of 0.1907 and a value of *p* of less than 0.0001, confirming the consistent and significant impact of ERI on the Intention to Quit.

In exploring the mediating variables, the analysis identified several noteworthy pathways. Notably, the primary predictors of Job Satisfaction were identified as Factor I: Integrity and Factor II: Confucian Ethos, followed by Factor IV: Moderation. For instance, Factor I: Integrity and Factor IV: Moderation demonstrated statistically significant effects on the Intention to Quit, with *p*-values of 0.0528 and 0.0317, respectively. A significant observation was that Job Satisfaction negatively influenced the Intention to Quit (coefficient = −0.3715, *p* < 0.0001), suggesting that increased Job Satisfaction could potentially reduce the tendency to quit. Detailed insights into these relationships are provided in Table 1.

**Table 1:** Pearson’s correlation matrix (*N* = 332).

Variable	1.	2.	3.	4.	5.	6.	7.	8.
1.Age	–							
2.Seniority	0.833***	–						
3.Effort-reward imbalance	0.036	−0.020	–					
4.CVS. Factor integrity	0.122*	0.191***	−0.154**	–				
5.CVS. Factor Confucian ethos	0.014	0.103	−0.352***	0.552***	–			
6.CVS. Factor loyalty	−0.052	0.048	−0.890***	0.225***	0.463***	–		
7.CVS. Factor moderation	−0.031	0.106	−0.314***	0.393***	0.581***	0.314***	–	
8.Job satisfaction	0.139*	0.226***	−0.171**	0.592***	0.636***	0.249***	0.455***	–
9.Intention to quit	−0.181***	−0.259***	0.496***	−0.249***	−0.383***	−0.485***	−0.238***	−0.519***

*p < 0.05, **p < 0.01, ***p < 0.001. CVS, Chinese Value Survey.

It is essential to acknowledge the variation in how Effort-Reward Imbalance indirectly affects the Intention to Quit via different mediators. The path involving Factor II: Confucian Ethos to Job Satisfaction and then to Intention to Quit (Beta = 0.0812) demonstrated a notably strong indirect effect, evidenced by a bootstrapped confidence interval that does not encompass zero. In contrast, the path from Factor I: Integrity through Job Satisfaction to Intention to Quit (Beta = 0.0286) also showed statistical significance, although its impact was relatively smaller. On the other hand, the path involving Factor III: Loyalty presented a confidence interval that included zero, suggesting that its indirect effect is not statistically significant.

### Descriptive statistics Pearson’s correlational analysis

In regard to the distribution of the data collected in our study, extensive statistical testing was conducted to determine its normality. Initial analyses were performed using the Shapiro–Wilk test, a reliable method for assessing normality in sample data. Additionally, we examined skewness and kurtosis values for each variable to ensure they fell within the acceptable range for normal distribution (−2 to +2 for skewness and −7 to +7 for kurtosis). Visual inspections of histograms, Q-Q plots, and box plots were also conducted as supplementary methods to assess the distribution of the data. These combined methods provided a comprehensive view of the data’s distribution, confirming that the assumption of normality was met for the key variables in our analysis. This confirmation of normal distribution is crucial for the validity of subsequent tests used in our study.

In relation to the key variables, it was observed that the teachers demonstrated a low propensity to leave their jobs, with an average intention to quit score of 6.23 and a standard deviation of 3.78. Their perceived effort-reward imbalance was also low, averaging at −17.37 with a standard deviation of 9.83.

Within the CVS, the highest score was recorded for Factor III, Loyalty, with a mean of 31.13 and a standard deviation of 7.91. In contrast, the lowest score was noted for Factor IV, Moderation, with a mean of 12.39 and a standard deviation of 3.04. The job satisfaction level among teachers was considered moderate, with a mean score of 28.31 and a standard deviation of 5.71.

As demonstrated in Table 2, there is a high and highly significant correlation between age and seniority (*p* < 0.001), indicating a robust positive association. On the other hand, the intention to quit shows a negative correlation with both age and seniority at *p* < 0.001. This suggests that younger individuals with lesser seniority are more inclined toward quitting. The factors within the CVS also revealed noteworthy correlations. A significantly high negative correlation exists between the Effort-Reward Imbalance and CVS Factor Loyalty, suggesting a strong inverse relationship. Job Satisfaction was found to have a strong positive correlation with CVS factors such as Factor I Integrity and Factor II Confucian Ethos, both significant at *p* < 0.001. These findings imply a close association between job satisfaction and these cultural values.

**Table 2:** Unstandardized coefficients.

Outcome variable						
Intention to quit						
Model summary						
*R*	*R*-sq	MSE	*F*	df1	df2	*p*
0.6784	0.4602	7.8730	46.1770	6.0000	325.0000	0.0000

Model						
	Coefficient	SE	*t*	*p*	LLCI	ULCI
Constant	16.1444	1.0997	14.6814	0.0000	13.9811	18.3078
Effort-reward imbalance	0.1675	0.0355	4.7145	0.0000	0.0976	0.2373
Factor I: integrity and tolerance	0.0687	0.0353	1.9434	0.0528	−0.0008	0.1382
Factor II: Confucian ethos	0.0111	0.0444	0.2494	0.8032	−0.0763	0.0985
Factor III: loyalty to ideals and humanity	−0.0112	0.0461	−0.2420	0.8090	−0.1019	0.0796
Factor IV: moderation and moral discipline	−0.1388	0.0643	2.1576	0.0317	0.0122	0.2654
Job satisfaction	−0.3715	0.0380	−9.7679	0.0000	−0.4464	−0.2967

## Discussion

This study primarily investigated the effect of ERI on the decision to leave one’s job among vocational educators in China, focusing on the intermediary roles of Confucian Values and job satisfaction in this context. The study’s foundational model’s robustness underscores the effectiveness of the analytical approach used. There was a notable direct correlation between Effort-Reward Imbalance and the desire to quit, consistent with prior studies highlighting the link between these imbalances and an employee’s likelihood of leaving an organization.

A key differentiator of this research is its in-depth analysis of the mediating influence of Confucian values, particularly focusing on aspects like Integrity, Confucian Ethos, and Moderation. The mediated effect via Confucian Ethos was especially strong, suggesting that embracing these values could help lessen the negative impact of Effort-Reward Imbalance. Given their emphasis on benevolence and equilibrium, employees who adhere to these values might tolerate imbalances more readily to preserve workplace harmony. Such an orientation could lead them to value non-material rewards, such as social support and positive workplace relationships, potentially enhancing job satisfaction and reducing the likelihood of quitting (Ong and Koh, 2021).

This discovery opens new pathways for cross-cultural research, examining the influence of traditional philosophies in contemporary workplace settings. The mediated effect via Integrity also held statistical significance, where concepts like filial piety might make employees more accepting of authority, reducing their inclination to voice concerns about effort-reward discrepancies (Schwartz and Bardi, 2001). A sense of righteousness and duty could encourage more effort despite limited rewards, maintaining job satisfaction. Integrity could drive individuals to adhere to ethical and professional standards, sustaining job satisfaction even amidst imbalances. Thus, Factor I could serve as a crucial mediator in softening the negative impacts of Effort-Reward Imbalance on job satisfaction and the intention to quit (Han, 2022).

Conversely, Factor III: Loyalty did not show a significant mediating role, differing from earlier studies that often highlighted loyalty’s importance in employee retention. The hypothesis was that loyalty denotes a deep commitment to one’s role and institution, which could make employees more tolerant of imbalances and less likely to quit. Ritual propriety, which involves respect for traditional practices and hierarchies, might foster a strong institutional belonging. Hence, Factor III’s values could act as mediators, encouraging employees to accept imbalances, maintaining job satisfaction and reducing quitting probabilities.

The lack of statistical significance in the pathway involving Loyalty calls for further exploration, questioning the relevance of traditional values in modern organizational contexts. The total impact of Effort-Reward Imbalance on the intention to quit was also significant, highlighting this factor’s extensive influence. Both direct and total effects corroborate the pivotal role of Effort-Reward Imbalance in shaping an employee’s intention to depart. This finding seems to contrast with the Confucian principle of ‘Zhongyong,’ or the Doctrine of the Mean, which advocates for balance and harmony, as the data indicates that an imbalance in effort and reward might lead to intentions to quit, opposing the Confucian ideal of balance (Tan, 2017).

Our study supports previous research indicating a significant direct effect of job stress on various Chinese workers, including soccer referees (Liu et al., 2022), emergency physicians (Jiang et al., 2022; Tian et al., 2022), and healthcare workers (Ge et al., 2021). This aligns with the broader ERI literature, which consistently points out the negative impacts of imbalances between efforts and rewards on work-related outcomes (Deng et al., 2021; Fei et al., 2023), demonstrating the ERI model’s wide applicability across different occupational contexts in China.

A unique aspect of our research is the examination of Confucian values as mediators in the relationship between ERI, job satisfaction, and the intention to quit. Our results confirm that Confucian values are significant in the stress-well-being relationship (Rochelle, 2019; Huo et al., 2020) and support previous findings on factors influencing employees’ decisions to stay with organizations (Li et al., 2017; Wong et al., 2019; Jiang et al., 2022).

In expanding the discussion of our findings, we emphasize the implications of integrating Confucian values into the ERI model. This approach provides a unique lens to examine how traditional philosophies can mitigate the challenges of modern workplace stressors. Our analysis suggests that Confucian values such as Integrity and Confucian Ethos play a significant role in shaping vocational educators’ responses to ERI. This insight not only contributes to a deeper understanding of cultural influences on job satisfaction and turnover intentions but also offers practical guidance for organizations operating in culturally diverse environments. By acknowledging and aligning with these cultural values, organizations can develop more effective strategies for employee engagement and retention, thereby enhancing overall workplace harmony and productivity.

Furthermore, our study opens avenues for future research to explore the intersection of traditional values and contemporary organizational dynamics. The nuanced interaction between Confucian values and ERI in the context of Chinese vocational education highlights the potential for similar investigations in other cultural settings. Such research can provide valuable insights for multinational corporations and global organizations, emphasizing the importance of cultural sensitivity in management practices. The incorporation of traditional cultural values into organizational behavior studies not only enriches the academic discourse but also has significant implications for global workforce management, particularly in terms of designing culturally relevant employee support programs and policies.

In essence, the Chain Mediation Model has shed light on the intricate interactions between Effort-Reward Imbalance and the intention to quit, mediated by Confucian values. These insights not only contribute to existing literature but also lay a foundation for future research exploring the intersection of traditional Eastern philosophies and contemporary organizational behavior.

### Limitations of the present study

While this research provides valuable insights, it also presents several limitations that must be acknowledged. Firstly, the sample is limited exclusively to Chinese vocational education teachers. This specificity restricts the broader applicability of the results, as it does not encompass the full spectrum of educational professionals. The findings may therefore not be fully representative or applicable to educators in different contexts or cultures.

Another limitation arises from the use of social media and email for participant recruitment, which could lead to self-selection bias. This recruitment method might have resulted in a sample with different professional perspectives or levels of technological proficiency compared to educators who are less engaged with these digital platforms. Consequently, this approach may have influenced the outcomes and possibly depersonalized some of the study evidence.

Additionally, the study focuses on a limited range of demographic factors, specifically age, gender, and organizational tenure. This narrow focus means that the research may not have fully explored other potential moderating influences that could significantly impact the findings. As a result, there could be other relevant demographic variables that were not considered, potentially limiting the depth and applicability of the conclusions drawn.

In terms of the research tools utilized, the use of the Turnover Intention Questionnaire (TIQ), initially created for nursing professionals, may not be entirely appropriate for vocational education teachers. Moreover, the internal consistency of the Factor IV: Moderation and Moral Discipline subscale in the Chinese Value Survey was marginally below the standard benchmark, indicating possible issues with this measurement (Bennett, 2021).

Finally, the cross-sectional nature of the study presents limitations in terms of design and temporal analysis. This approach restricts the ability to draw causal conclusions and to understand the evolution and dynamics of the observed phenomena over time. The findings must, therefore, be interpreted with caution, as they reflect a snapshot in time rather than a longitudinal progression.

#### Future advancements

As we look forward to the future of research in this area, several advancements emerge from our current study’s findings. The integration of traditional Confucian values within the Effort-Reward Imbalance (ERI) model has opened new pathways for understanding workplace dynamics in culturally rich environments. Future research should continue to explore this intersection, examining how traditional philosophies can be harmonized with contemporary organizational practices across various cultural contexts.

There is significant potential for expanding this research beyond the realm of Chinese vocational education. Investigating the impact of cultural values on workplace stress and employee satisfaction in different cultural settings and industries can provide a more comprehensive understanding of global workplace dynamics. This could include comparative studies across different countries or within multinational corporations, offering insights into how cultural values influence employee behavior and organizational outcomes in a globalized economy.

Another advancement lies in the application of our findings to practical organizational strategies. Our study suggests that understanding and aligning with employees’ cultural values can be a crucial element in enhancing job satisfaction and reducing turnover intentions. Future research can focus on how organizations can effectively integrate these insights into their human resource policies and management practices. This could involve developing culturally sensitive training programs, employee engagement initiatives, and leadership development strategies that respect and leverage the cultural values of the workforce.

Lastly, technological advancements offer new opportunities for collecting and analyzing data in this field. The use of sophisticated data analytics tools and artificial intelligence can provide deeper insights into the complex interplay of cultural values, workplace stress, and employee behavior. Future studies might employ these technologies to conduct more nuanced analyses, potentially uncovering patterns and correlations that were not previously apparent.

### Suggestions for intervention

Based on the current research findings, a number of intervention strategies are suggested to promote organizational wellbeing among vocational education teachers in China. Initially, it is crucial for institutions to re-evaluate and possibly restructure their Effort-Reward balance for educators. The compelling evidence from this study showing a direct impact of Effort-Reward Imbalance on quitting intentions underscores the necessity for educational institutions to revisit their reward systems. Adjustments could include a mix of monetary and non-monetary incentives, such as performance-based bonuses, advancement opportunities, or acknowledgment and affirmative feedback, which are integral in making educators feel appreciated for their contributions (Zhang, 2022).

Furthermore, considering the notable intermediary role of Confucian Values like Integrity and Confucian Ethos in job satisfaction, it would be beneficial to integrate these values into professional development initiatives (Kong et al., 2022). Such programs should aim at cultivating a workplace atmosphere aligned with these traditional cultural values, which could enhance job satisfaction and potentially decrease the likelihood of resignation.

Additionally, the research indicates that increased job satisfaction may reduce the inclination to leave (Ge et al., 2021; Cheng et al., 2022). Thus, it would be advantageous for educational organizations to conduct regular assessments of job satisfaction levels among their educators and implement necessary corrective measures. This could encompass enhancements in work conditions, more opportunities for professional advancement, or support for mental health, considering the demanding nature of the teaching profession (Pan et al., 2015; Li et al., 2017).

## Conclusion

To summarize, the complex and culturally specific role that Confucian values play in mediating the relationship between Effort-Reward Imbalance, job satisfaction, and the likelihood of quitting among vocational education teachers in China is a multifaceted and significant aspect. These values, when incorporated in the work environment, act as a mitigating factor, shaping the way educators perceive and react to the stress stemming from Effort-Reward Imbalance. Grasping the dynamics between these traditional values and occupational stress is crucial in formulating effective support and retention strategies for vocational education teachers, thereby improving the overall educational experience for young students.

## Data availability statement

The raw data supporting the conclusions of this article will be made available by the authors, without undue reservation.

## Ethics statement

The studies involving humans were approved by Ethics Committee of Chongqing City Management College. The studies were conducted in accordance with the local legislation and institutional requirements. The participants provided their written informed consent to participate in this study.

## Author contributions

GW: Writing – original draft, Writing – review & editing. JS: Writing – original draft, Writing – review & editing.
